# Development and Preliminary Application of Multiplex Loop-Mediated Isothermal Amplification Coupled With Lateral Flow Biosensor for Detection of *Mycobacterium tuberculosis Complex*

**DOI:** 10.3389/fcimb.2021.666492

**Published:** 2021-04-27

**Authors:** Xingyun Wang, Guirong Wang, Yacui Wang, Shuting Quan, Hui Qi, Lin Sun, Chen Shen, Hairong Huang, Weiwei Jiao, Adong Shen

**Affiliations:** ^1^ Key Laboratory of Major Diseases in Children, Ministry of Education, National Key Discipline of Pediatrics (Capital Medical University), National Clinical Research Center for Respiratory Diseases, Beijing Key Laboratory of Pediatric Respiratory Infection Diseases, Beijing Pediatric Research Institute, Beijing Children’s Hospital, Capital Medical University, Beijing, China; ^2^ National Tuberculosis Clinical Laboratory, Beijing Key Laboratory for Drug Resistance Tuberculosis Research, Beijing Tuberculosis and Thoracic Tumor Research Institute, Beijing Chest Hospital, Capital Medical University, Beijing, China

**Keywords:** *Mycobacterium tuberculosis complex*, loop-mediated isothermal amplification, lateral flow biosensor, LAMP-LFB, tuberculosis

## Abstract

The aim of this study was to develop a simple and reliable method to detect *Mycobacterium tuberculosis complex* (MTBC) and verify its clinical application preliminarily. A loop-mediated isothermal amplification method coupled with lateral flow biosensor (LAMP-LFB) assay, was developed and evaluated for detection of MTBC. Two sets of primers, which targeted IS*6110* and IS*1081* sequences of MTBC, were designed for establishment of multiplex LAMP-LFB assay. The amplicons were labelled with biotin and fluorescein isothiocyanate (FITC) by adding FITC labelled primer and biotin-14-dATP and biotin-14-dCTP and could be visualized using LFB. The optimal reaction conditions of multiplex LAMP-LFB assay confirmed were 66°C for 50 min. The analytical sensitivity of multiplex LAMP-LFB is 10 fg of genomic templates using pure culture, and no cross-reactivity with other common bacteria and non-tuberculous mycobacteria strains was obtained. A total of 143 clinical samples collected from 100 TB patients (62 definite TB cases and 38 probable TB cases) and 43 non-TB patients were used for evaluating the feasibility of multiplex LAMP-LFB assay. The multiplex LAMP-LFB (82.0%, 82/100) showed higher sensitivity than culture (47.0%, 47/100, P < 0.001) and Xpert MTB/RIF (54.0%, 54/100, P < 0.001). Importantly, the multiplex LAMP-LFB assay detected additional 28 probable TB cases, which increased the percentage of definite TB cases from 62.0% (62/100) to 90.0% (90/100). The specificity of multiplex LAMP-LFB assay in patients without TB was 97.7% (42/43). Therefore, multiplex LAMP-LFB assay is a simple, reliable, and sensitive method for MTBC detection, especially in probable TB cases and resource limited settings.

## Introduction

Tuberculosis (TB) is the leading cause of death by single infectious disease worldwide. However, up to 29% of estimated incident cases failed to be diagnosed and reported (World-Health-Organization, 2020). Rapid and reliable diagnosis of TB is a prerequisite for successful treatment, thus alternative accurate screening and diagnostic techniques are urgently needed for sensitive, effective and specific detection of MTBC.

Traditionally, smear microscopy is the routine tool for MTBC detection in resource-limited regions for its simplicity and low cost (Steingart et al., 2006). However, it lacks sensitivity and often requires more than a single visit of a patient to be completed. Mycobacterial culture is still the gold standard for TB diagnosis due to its high sensitivity and specificity. But it is time-consuming and technically demanding, which limits its wide application at reference centers. In recent years, polymerase chain reaction (PCR)-based techniques (such as conventional PCR, real-time PCR, multiplex PCR) are attractive and promising for detection of MTBC (Lekhak et al., 2016; Kabir et al., 2018; Molaudzi and Molepo, 2019; Nyaruaba et al., 2019), but they need expensive laboratory instruments and complicated technical skill which make them inaccessible in resource-poor areas.

Loop-mediated isothermal amplification (LAMP) is a promising technique, which can eliminate the shortcomings mentioned above. Moreover, it has been endorsed by World Health Organization for MTBC detection in 2016 (Notomi et al., 2000; World-Health-Organization, 2016). The results of previously reported LAMP-based assays have usually been determined by agarose gel electrophoresis, colorimetric indicator (SYBR green I or calcein dyes) and real-time turbidity. Particularly, analysis of LAMP results using agarose gel electrophoresis requires complicated operation and time-consuming steps.; using colorimetric indicator to detect LAMP amplification products is potentially subjective, and sometimes produces ambiguous judgment; and detection of LAMP products using real-time turbidity needs expensive optical instrument (Zhang et al., 2014). Recently, lateral flow biosensor (LFB) combined with LAMP has been used for detection of many pathogens (Arunrut et al., 2010; Mori et al., 2013; Wang et al., 2017). It owns the advantages of rapid, sensitive and objective interpretation of the results. However, studies on LAMP-LFB for MTBC detection are limited (Kaewphinit et al., 2013; Roskos et al., 2013; Joon et al., 2019).

In this study, we designed a LAMP assay simultaneously targeting two specific genes IS6110 and IS1081, and combined with nanoparticles-based lateral flow biosensor (LAMP-LFB) for rapid, visible, sensitive and reliable diagnosis of MTBC. The sensitivity, specificity and feasibility of the assay were preliminarily evaluated using pure cultures and clinical samples.

## Materials and Methods

### Reagents and Apparatus

The Isothermal amplification kit (includes *Bst*2.0 DNA polymerase and reaction buffer), biotin-14-dATP, biotin-14-dCTP were purchased from HuiDexin Biotechnology Co., Ltd (Tianjin, China). Dye streptavidin-coated polymer nanoparticles (SA-DNPs) were bought from the Resenbio. Co., Ltd. (XiAn, China). The fluorescein isothiocyanate antibody (anti-FITC) and biotinylated bovine serum albumin (biotin-BSA) were bought from Abcam. Co., Ltd. (Shanghai, China). Backing card, sample pad, conjugate pad, nitrocellulose membrane (NC) and absorbent pad were purchased from the Jie-Yi Biotechnology. Co., Ltd. (Shanghai, China). QIAamp DNA Mini Kit was purchased from Qiagen Co., ltd (Beijing, China). Glass bead-based kits (CapitalBio Co.) were bought from BoaoJingxin Biotechnology Co., Ltd (Chengdu, China).

### Primer Design

**Figure 1:**
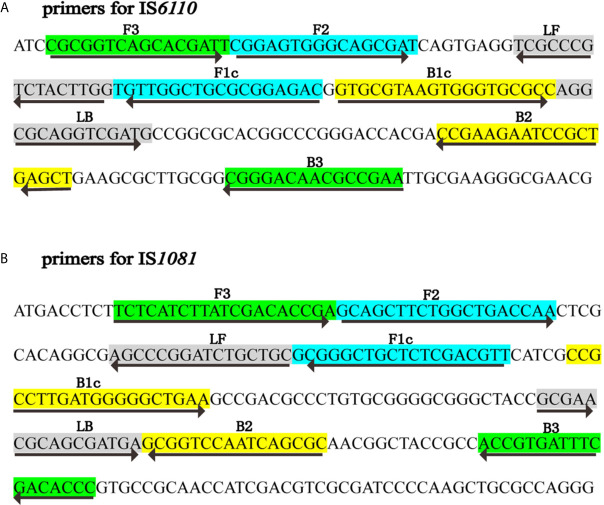
Primers specific to IS*6110* and IS*1081* of MTB used for LAMP-LFB assay. Sequence and locations of insertion sequences IS*6110* and IS*1081* used to design LAMP primers. The nucleotide sequences of IS*6110* **(A)** and IS*1081* **(B)** are shown. Primer FIP included F2 and F1c; Primer BIP included B1c and B2. The direction of arrows indicated the primer from 5’ to 3’.

Two sets of LAMP primers, which are targeted specific for IS*6110* and IS*1081*, respectively, were designed using Primer Explorer V5 (http://primerexplorer.jp/e) according to the principles described by Notomi et al. (2000). Each primer set consisted of two inner primers (FIP, BIP), two outer primers (F3, B3) and two loop primers (LF, LB). To achieve the LFB detection, the FIP primer was labeled with FITC at the 5’ end, and was named as FIP*. The specificity of primers was confirmed by sequence alignment analysis using the basic local alignment search tool (BLAST). The locations and sequences of primers were shown in **Figure 1** and **Table 1**. Primers used in this study were synthesized by TianyiHuiyuan Biotechnology Co., Ltd (Beijing, China).

**Table 1:** The sequences of primer sets used in this study.

Primer name	Sequences (5′-3′) and modifications	Target
**F3-*6110***	CGCGGTCAGCACGATT	
**B3-*6110***	TTCGGCGTTGTCCCG	
**FIP-*6110***	GTCTCCGCGCAGCCAACACGGAGTGGGCAGCGAT	**IS*6110***
FIP*-*6110*	5’-FITC-GTCTCCGCGCAGCCAACACGGAGTGGGCAGCGAT-3’	
**BIP-*6110***	GTGCGTAAGTGGGTGCGCCAGCTCAGCGGATTCTTCGG	
**LF-*6110***	CCAAGTAGACGGGCGA	
**LB-*6110***	AGGCGCAGGTCGATG	
**F3-*1081***	TCTCATCTTATCGACACCGA	
**B3-*1081***	GGGTGTCGAAATCACGGT	
**FIP-*1081***	AACGTCGAGAGCAGCCCGCGCAGCTTCTGGCTGACCAA	**IS*1081***
FIP*-*1081*	5’-FITC-AACGTCGAGAGCAGCCCGCGCAGCTTCTGGCTGACCAA-3’	
**BIP-*1081***	CCGCCTTGATGGGGGCTGAAGCGCTGATTGGACCGC	
**LF-*1081***	GCAGCAGATCCGGGCT	
**LB-*1081***	GCGAACGCAGCGATGA	

F3, Forward outer primer; B3, Backward outer primer; FIP, Forward inner primer; BIP, Backward inner primer; LF, Forward loop primer; LB, Backward loop primer; FIP*, Forward inner primer labeled with FITC at 5’ end.

### Bacterial Strains and Genomic DNA Extraction

A total of 47 bacterial strains, including 1 MTBC reference strain H37Rv, 1 *M. bovis* strain BCG, 12 MTBC clinical strains isolated from TB patients, 25 non-mycobacteria and 8 non-tuberculous mycobacteria (NTM) strains were used in this study (**Table 2**). Genomic DNA was extracted using QIAamp DNA Mini Kit according to the manufacturer’s instructions. The amount of DNA was quantified on NanoDrop ND-1000 (Calibre, Beijing, China).

**Table 2:** Bacteria strains used in this study.

Bacteria	Strain no. (source of strains)^a^	No. of strains	LAMP-LFB result^b^
*M. tuberculosis*	H37RV	1	P
*M. bovis BCG* *M. tuberculosis*	BCG Isolated strains (BCH)	1 12	P P
*M. fortuitum*	Isolated strain (BCH-NTCL)	1	N
*M. abscessus*	Isolated strain (BCH-NTCL)	1	N
*M. avium*	Isolated strain (BCH-NTCL)	1	N
*M. intracellulare*	Isolated strain (BCH-NTCL)	1	N
*M. malmoense*	Isolated strain (BCH-NTCL)	1	N
*M. gordonae*	Isolated strain (BCH-NTCL)	1	N
*M. marcellus*	Isolated strain (BCH-NTCL)	1	N
*M. swine*	Isolated strain (BCH-NTCL)	1	N
*Klebsiella pneumoniae*	Isolated strains (BCH)	1	N
*Pseudomonas aeruginosa*	Isolated strains (BCH)	1	N
*Streptococcus pneumoniae*	Isolated strains (BCH)	1	N
*Listeria monocytogenes*	Isolated strains (BCH)	1	N
*Staphylococcus epidermidis*	Isolated strains (BCH)	1	N
*Staphylococcus aureus*	Isolated strains (BCH)	1	N
*Staphylococcus saprophyticus*	Isolated strains (BCH)	1	N
*Bacillus cereus*	Isolated strains (BCH)	1	N
*Bntorobater sakazakii*	Isolated strains (BCH)	1	N
*Campylobacter jejuni*	Isolated strains (BCH)	1	N
*Escherichia coli*	Isolated strains (BCH)	1	N
*Plesiomonas shigelloides*	Isolated strains (BCH)	1	N
*Salmonella enterica*	Isolated strains (BCH)	1	N
*Shigella flexneri*	Isolated strains (BCH)	1	N
*Vibrio parahaemolyticus*	Isolated strains (BCH)	1	N
*Vibrio vulnificus*	Isolated strains (BCH)	1	N
*Enterococcus faecalis*	Isolated strains (BCH)	1	N
*Enterococcus faecium*	Isolated strains (BCH)	1	N
*Enterococcus hirae*	Isolated strains (BCH)	1	N
*Bacillus pertussis*	Isolated strains (BCH)	1	N
*Acinetobacter baumannii*	Isolated strains (BCH)	1	N
*Haemophilus influenzae*	Isolated strains (BCH)	1	N
*Stenotrophomonas maltophilia*	Isolated strains (BCH)	1	N
*Mycoplasma pneumoniae*	Isolated strains (BCH)	1	N
*Streptococcus agalactiae*	Isolated strains (BCH)	1	N

a BCH, Beijing Children’s Hospital; BCH-NTCL, National Tuberculosis Clinical Laboratory, Beijing Chest Hospital.

b P, positive; N, negative.

### Singlex and Multiplex LAMP Assay

The singlex standard LAMP reaction for IS*6110* or IS*1081* was performed for verifying the suitability of primer sets. Total 25 μl mixtures comprised of 0.8 µM each FIP* and FIP primers, 1.6 µM BIP primers, 0.8 µM each LF and LB primer, 0.4 µM each F3 and B3 primers, 12.5 µl 2×reaction buffer, 1 µl Bst DNA polymerase (8 U), 1 µl of 50 nM biotin-14-dATP, 1 µl of 50 nM biotin-14-dCTP and 1 µl DNA template. Biotin-14-dATP and biotin-14-dCTP were added to the reaction mixture to label the LAMP products with biotin. Genomic DNA of H37Rv (100 fg) was used as positive control, *Streptococcus pneumoniae* (1 ng) and *Escherichia coli* (1 ng) were used as negative control, and double distilled water (DW) as blank control. The mixtures were incubated at 66°C for 50 min, and then the amplification products were detected by LFB and agarose gel electrophoresis.

The multiplex LAMP was also carried out in a 25 µl mixture comprised of 0.4 µM each of FIP*-*6110*, FIP-*6110*, LF-*6110*, LB-*6110*, FIP*-*1081*, FIP-*1081*, LF-*1081* and LB-*1081*, 0.8 µM of each BIP-*6110* and BIP-*1081*, 0.2 µM of each F3-*6110*, B3-*6110*, F3-*1081* and B3-*1081* primers, 12.5 µl 2×reaction buffer, 1 µl Bst DNA polymerase (8 U), 1 µl of 50 nM biotin-14-dATP, 1 µl of 50 nM biotin-14-dCTP and DNA template (1 µl for bacterial strains, 5 µl for clinical specimens). The multiplex LAMP reactions were performed at 66°C for 50 min.

### LFB Assay

In the LAMP reaction system, FIP* (FITC labeled primer), biotin-14-dATP and biotin-14-dCTP were added into the amplification mixture. After LAMP reaction, the amplicons were simultaneously labeled with FITC and biotins, and can be detected by LFB (Wang et al., 2017). The LFB was prepared and performed as previous study (Wang et al., 2017). Briefly, four components (sample pad, conjugate pad, NC membrane and absorbent pad) were assembled together on a backing card. SA-DNPs were impregnated on the conjugate pad, capture reagents anti-FITC antibody and biotin-BSA were immobilized at the test line (TL) and control line (CL) of NC membrane, respectively. When detected using LFB, 0.5 µl LAMP amplification products and 70 µl running buffer were added to the immersion pad, biotin was integrated with SA-DNPs, FITC was captured by anti-FITC antibody, and then FITC/LAMP/SA-DNPs complexes were indicated by crimson red lines. The remaining SA-DNPs were transferred and combined with biotin-BSA immobilized at CL for quality monitoring of the LFB. Two red lines could be seen at TL and CL when LAMP result was positive. Only one red line appeared at CL, indicating the negative results.

### Optimization of LAMP Reaction Temperature

The IS*6110*-LAMP and IS*1081*-LAMP reaction mixtures were performed at the temperature ranging from 61°C to 68°C at 1°C intervals for 1 h to determine the optimal reaction temperature. The amplifications were monitored by Loopamp LA-320C Realtime Turbidimeter (Eiken, Janpan). Genomic DNA of *Streptococcus pneumoniae* (1 ng) and *Escherichia coli* (1 ng) was used as negative control. The IS*6110*-LAMP and IS*1081*-LAMP reactions were performed three times each.

### Sensitivity and Specificity of the LAMP-LFB Assay

To evaluate the analytical sensitivity of IS*6110*-LAMP-LFB, IS*1081*-LAMP-LFB and multiplex LAMP-LFB assays for MTBC identification, serially diluted (1 ng, 100 pg, 10 pg, 1 pg, 100 fg, 10 fg, 1 fg) genomic DNA of H37Rv were prepared. The last positive dilution was considered as the limit of the detection (LoD). Amplification products were monitored by LFB, and further confirmed by agarose gel electrophoresis. Each dilution was tested three times to confirm the LoD of LAMP-LFB assay.

To assess the analytical specificity of multiplex LAMP-LFB assay for MTBC detection, reactions were performed under the conditions depicted above with DNA templates extracted from 1 MTBC reference strain H37Rv, 12 MTBC strains isolated from TB patients, 1 BCG strain, 25 non-mycobacteria and 8 NTM strains (**Table 2**).

### Optimization of LAMP Reaction Time

To optimize the reaction time of multiplex LAMP assay, the assays were conducted at optimal temperature for 30 min, 40 min, 50 min, and 60 min with template at the LoD level. The reactions were conducted three times.

### Application of Multiplex LAMP-LFB Assay in Clinical Specimens

To further evaluate the applicability of LAMP-LFB assay in clinical samples, 143 suspected pulmonary TB patients were enrolled from Beijing Chest Hospital during January 2, 2019 to January 31, 2019. All the patients were subjected to culture, Xpert MTB/RIF and multiplex LAMP-LFB assay.

Patients were divided into three groups according to the composite reference standard, which was composed of clinical, laboratory, histopathologic and radiologic findings and follow-up data, proposed by Chinese Pulmonary Tuberculosis Diagnosis Criteria WS288-217 (National-Health-and-Family-Planning-commission-of-the-People’s-Republic-of-China, 2017). (i) Definite TB patients: represented by positive bacteriological examinations using smear microscopy, culture, molecular biology methods, or histopathological change. (ii) Probable TB patients: radiographic evidence that consistent with TB and at least one of the following: symptoms of TB, positive tuberculin skin test, or interferon-γ release test results, histopathological or bronchoscopic examination consistent with TB. (iii) non-TB patients: diagnosed as other diseases and the patients improved without anti-TB treatment.

Approximately 1 ml of sputum was used for each test. Culture was carried out using BACTEC 960 system (BD Microbial system, Sparks, MD, USA). Xpert MTB/RIF assay (Cepheid, Sunnyvale, CA, USA) was performed according to the manufacturer’s guidelines. For multiplex LAMP-LFB assay, 1 ml of sputum was digested with adding equal volume of 4% NaOH, and glass bead-based kit (CapitalBio Co.) was used to extract the genomic DNA. 5 µl of DNA templates extracted from sputum were added into the LAMP reaction mixtures, and the results were detected by LFB.

Our study was approved by Ethical committee of Beijing Chest Hospital and Beijing Children’s Hospital. Informed consent has been obtained from all enrolled patients.

### Statistical Analysis

The performance of multiplex LAMP-LFB was evaluated by using bacteriologic results and clinical evidence as composite reference standard. χ^2^ test or Fisher’s exact test was used to compare sensitivity differences between multiplex LAMP-LFB, Xpert MTB/RIF and culture. SPSS 23.0 software was used to do statistical analysis, and *P* < 0.05 was considered statistically significant.

## Results

### Confirmation and Detection of Amplification of LAMP Assay

**Figure 2:**
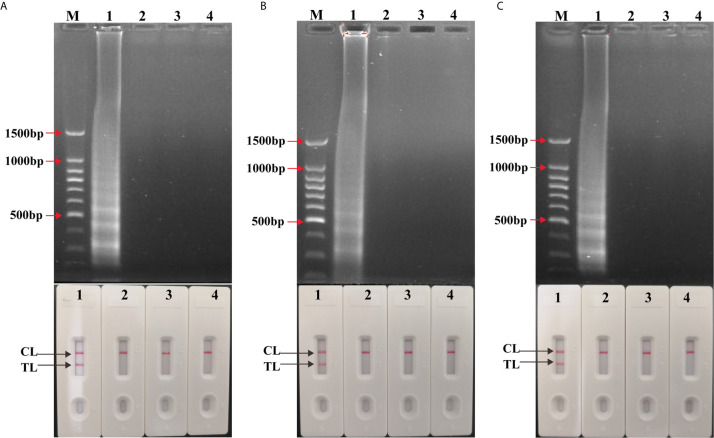
Confirmation and detection of singlex LAMP and multiplex LAMP products. **(A)** Confirmation and detection the amplicons of IS*6110*-LAMP and **(B)** IS*1081*-LAMP and **(C)** multiplex LAMP. Top: 1.5% agarose gel electrophoresis using for amplification products analysis. Bottom: LFB applied for visual detection of amplicons analysis. Biosensors/lanes 1-4: positive products of H37Rv, *Streptococcus pneumoniae* and *Escherichia coli* as negative control, DW as blank control.

To validate the availability of two LAMP primer sets, singlex LAMP and multiplex LAMP reactions were conducted using H37Rv as positive control, *Streptococcus pneumoniae* and *Escherichia coli* as negative control, DW as blank control (**Figure 2**). The LAMP amplicons were visualized using LFB, and further confirmed by agarose gel electrophoresis. As shown in LFB, there were two red lines (TL and CL) in positive tubes and only one red line (CL) in negative and blank controls. Many different sizes of bands were seen in positive control using agarose gel electrophoresis, but not in negative and blank controls. The results demonstrated that the two sets of LAMP primers were able to specifically identify MTBC synchronously by targeting IS*6110* and IS*1081*.

### The Optimal Reaction Temperature of LAMP Assay

**Figure 3:**
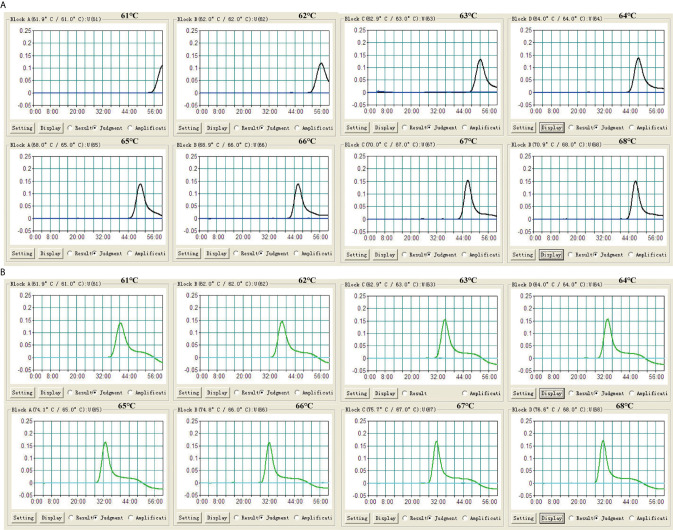
Optimization of reaction temperature for LAMP assay. The LAMP reactions were monitored by real-time turbidimeter. **(A)** IS*6110*-LAMP. **(B)** IS*1081*-LAMP. Turbidity of >0.1 was deemed to be positive. Eight kinetic graphs (1-8) were acquired at different temperatures (61°C to 68°C, 1°C intervals) with 100 fg of H37Rv DNA template per reaction.

To assess the optimal reaction temperature of LAMP assays, IS*6110*-LAMP and IS*1081*-LAMP reactions were carried out at temperatures from 61°C to 68°C with an interval of 1°C. According to the real-time turbidimeter, the optimum amplification temperature was 66°C-67°C for the IS*6110*-LAMP (**Figure 3A**), and 65°C-68°C for IS*1081*-LAMP (**Figure 3B**), respectively. Given this, 66°C was chosen as the reaction temperature in subsequent LAMP-based experiments.

### Sensitivity of Single and Multiplex LAMP-LFB Assay

**Figure 4:**
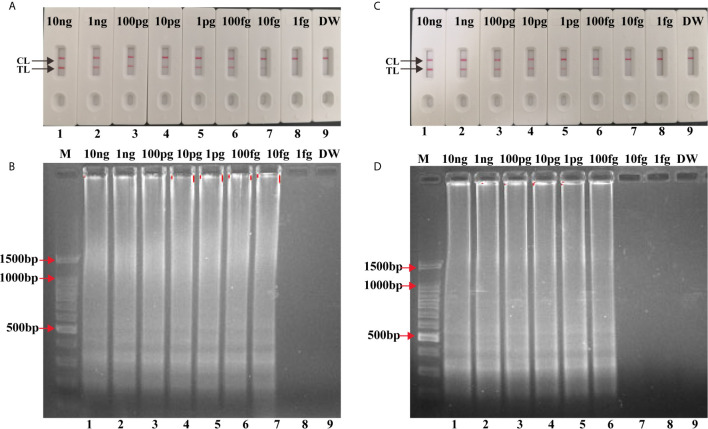
Analytical Sensitivity of singlex LAMP-LFB assay. Serial dilutions (10 ng, 1 ng, 100 pg, 10 pg, 1 pg, 100 fg, 10 fg, 1 fg) of genomic DNA of H37Rv were used for sensitivity determination of IS*6110*-LAMP **(A, B)** and IS*1081*-LAMP **(C, D)** assay. **(A, C)** Visual results of LFB for LAMP assay. **(B, D)** 1.5% agarose gel electrophoresis applied for LAMP products. Biosensors/lanes 1-9: H37Rv genomic DNA (10 ng, 1 ng, 100 pg, 10 pg, 1 pg, 100 fg, 10 fg, 1 fg), DW.

**Figure 5:**
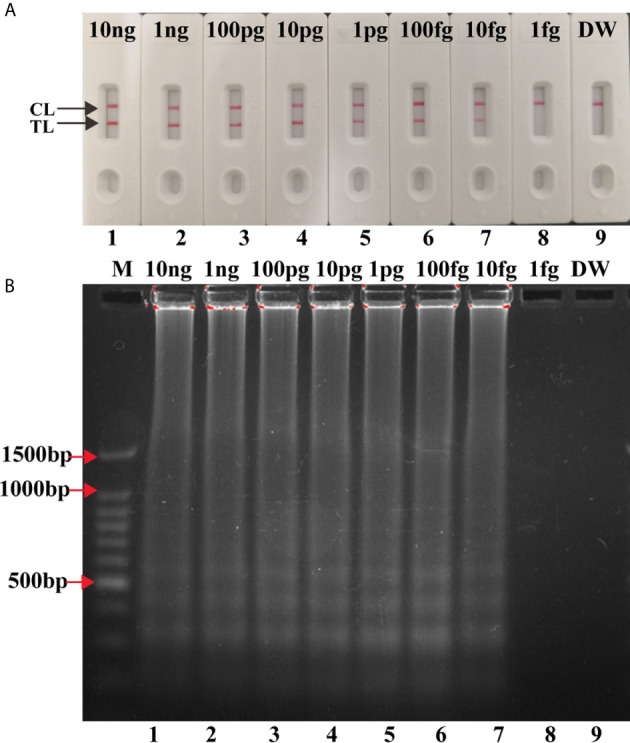
Analytical Sensitivity of multiplex LAMP-LFB assay. Two sets of primers targeting IS*6110* and IS*1081* were simultaneously added to one reaction tube to evaluate the sensitivity of LAMP assay. Two methods were used for amplification products analysis **(A)** LFB, **(B)** 1.5% agarose gel electrophoresis. Numbers 1-9: H37Rv genomic DNA (10 ng, 1 ng, 100 pg, 10 pg, 1 pg, 100 fg, 10 fg, 1 fg), DW.

Serial dilutions (10 ng to 1 fg) of H37Rv genomic DNA were used for LAMP reactions. As shown in **Figure 4A**, the LoD of IS*6110*-LAMP was 10 fg analyzed by LFB, which was consistent with agarose gel electrophoresis analysis (**Figure 4B**). The LoD of IS*1081*-LAMP for MTBC detection was 100 fg monitored by LFB (**Figure 4C**), which was also consistent with agarose gel electrophoresis analysis (**Figure 4D**). The same dilutions (10 ng to 1 fg) of H37Rv genomic DNA were further used for multiplex LAMP-LFB reactions. As shown in **Figure 5**, the LoD was 10 fg of templates per reaction for MTBC detection monitored by LFB and agarose gel electrophoresis.

### The Optimal Reaction Time for Multiplex LAMP-LFB Assay

**Figure 6:**
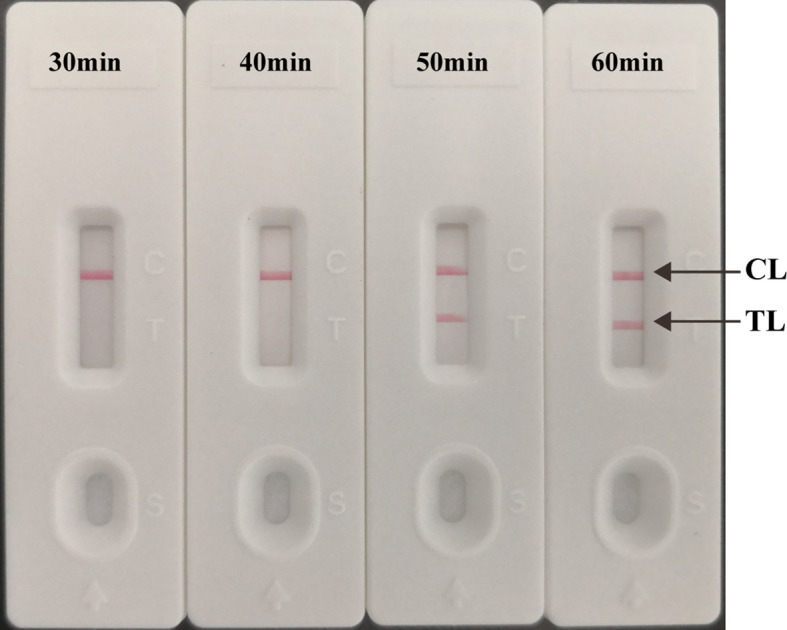
Optimized reaction Time for multiplex LAMP-LFB Assay. The LAMP reactions with template at the LoD level (10 fg) were amplified at 30 min, 40 min, 50 min, and 60 min at the optimal temperature (66°C).

The multiplex LAMP-LFB assays were conducted at 66°C using 10 fg H37Rv genomic DNA for 30 min, 40 min, 50 min, and 60 min, respectively. Two red lines (TL and CL) were seen when the multiplex LAMP assay lasted 50 min (**Figure 6**). Thus, 50 min was used as the optimal duration time for. Hence, the whole procedure, including DNA preparation (15 min), LAMP reaction (50 min), and result interpretation (2 min), could be accomplished within 70 min.

### Specificity of Multiplex LAMP-LFB Assay

**Figure 7:**
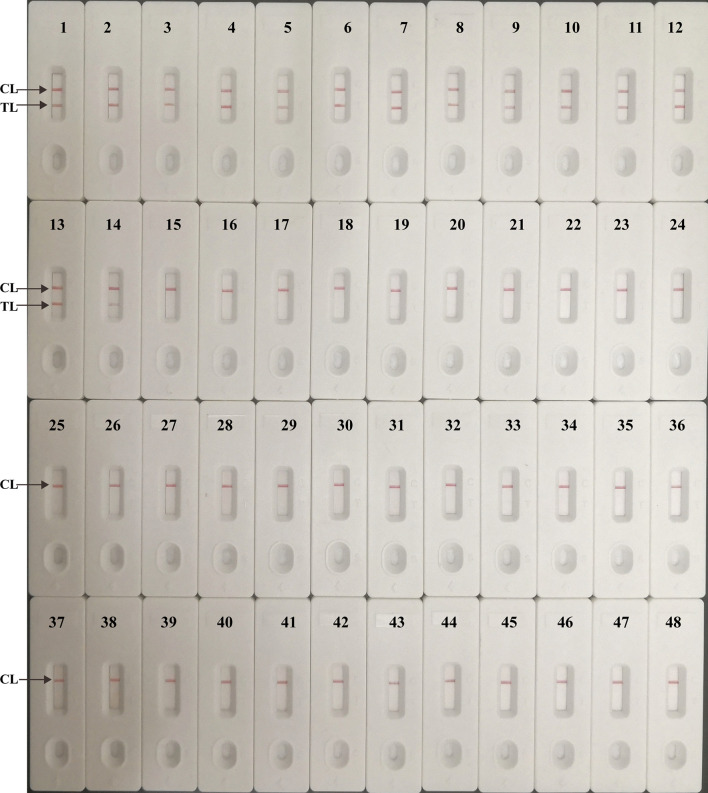
Analytical specificity of multiplex LAMP-LFB assay. The multiplex LAMP assays were carried out using various genomic DNA templates, and were monitored by LFB. Biosensor 1, H37Rv; Biosensor 2, BCG; Biosensors 3-14, 12 MTB strains isolated from clinical patients; Biosensors 15-22, 8 different NTM strains (details shown in **Table 2**)*;* Biosensor 23-47, 25 non-mycobacteria bacteria strains (details shown in **Table 2**); Biosensor 48, DW.

Genomic templates extracted from H37Rv, BCG, 12 MTBC clinical strains, 8 NTM isolates and 25 non-mycobacteria pathogens were analyzed to validate the specificity of multiplex LAMP-LFB assay. Positive results were only obtained from MTBC strains, and two red lines (TL and CL) simultaneously appeared on the biosensor. However, only one red band (CL) was shown in non-MTBC strains, which indicated that multiplex LAMP assay has a high specificity (100%) for MTBC detection (**Figure 7**).

### Application of Multiplex LAMP-LFB Assay in Clinical Samples

Total 143 patients were enrolled in the study, including 100 TB patients (62 definite TB cases, 38 probable TB cases) and 43 patients without TB. In the 100 TB patients, males accounted for 75.0% (75/100), the average age is 47.5 years old. The 43 patients without TB included 37 pneumonia cases, 4 malignant tumor cases and 2 bronchiectasis cases. There are 25 males (58.1%, 25/43) in the non-TB group, and the average age is 55.3 years old.

The performance comparison for MTBC detection showed that multiplex LAMP-LFB (82.0%, 82/100) produced a higher sensitivity than culture (47.0%, 47/100, P<0.001) and Xpert MTB/RIF (54.0%, 54/100, P<0.001) (**Table 3**) in the total TB patients. Multiplex LAMP-LFB (91.5%, 43/47) demonstrated higher sensitivity than Xpert MTB/RIF (83.0%, 39/47) among culture-positive cases. Especially, multiplex LAMP-LFB assay detected 73.7% (28/38) of probable TB cases. When the multiplex LAMP-LFB assay results were integrated into the composite reference standard, the percentage of definite patients showed an obvious increase from 62.0% (62/100) to 90.0% (90/100). In 43 patients without TB, none was positive using culture and Xpert MTB/RIF. One patient with pneumonia was positive using multiplex LAMP-LFB assay. Thus, the specificity of culture, Xpert MTB/RIF and LAMP-LFB were 100% (43/43), 100% (43/43) and 97.7% (42/43), respectively.

**Table 3:** Performance of different methods for TB diagnosis.

Patient Group	Culturen/N (%)	Xpertn/N (%)	Multiplex LAMP-LFBn/N (%)
Definite TB			
Sensitivity	47/62 (75.8)	54/62 (87.1)	54/62 (87.1)
Specificity	43/43 (100)	43/43 (100)	42/43 (97.7)
PPV	47/47 (100)	54/54 (100)	54/55 (98.2)
NPV	43/58 (74.1)	43/51 (84.3)	42/50(84.0)
Probable TB	–	–	
Sensitivity	–	–	28/38 (73.7)
Specificity	–	–	42/43 (97.7)
PPV	–	–	28/29 (96.6)
NPV	–	–	42/52 (80.8)
TB Total	–	–	
Sensitivity	47/100 (47.0)^a^	54/100 (54.0)^a^	82/100 (82.0)
Specificity	43/43 (100)	43/43 (100)	42/43 (97.7)
PPV	47/47 (100)	54/54 (100)	82/83 (98.8)
NPV	43/96 (44.8)	43/89 (48.3)	42/60 (70.0)

NPV, negative predictive value; PPV, positive predictive value.

a Statistically significant (P < 0.001) when compared with multiplex LAMP-LFB.

## Discussion

TB is a severe disease with high infectiousness, thus, speedy diagnosis and treatment are essential to control TB. Here, we designed a novel LAMP-LFB assay for rapid diagnosis of MTBC, which was proved to be a simple method with high sensitivity and specificity. The whole process, including template preparation (15 min), isothermal amplification (50 min) and LFB visual detection (2 min), can be completed within 70 min.

In this report, LFB was used for rapid, objective and visible analysis of LAMP results. Compared with other analysis methods (i.e., agarose gel electrophoresis, turbidity and colorimetric indicator) used in the previous reports (Pandey et al., 2008; Rodríguez-García et al., 2018; Thapa et al., 2019), the biosensor exhibited its superiority on rapid results, simple operation, and ease of use. Most importantly, using LFB to detect the MTBC-LAMP results was able to eliminate the use of additional procedure, expensive optical instrument instruments, and special reagent. Hence, the novel LAMP-LFB method was more suitable for reliable and convenient detection of target pathogens.

Our multiplex LAMP-LFB assay simultaneously detect two targets (IS*6110* and IS*1081*), which have multiple copies and exclusively exist in MTBC. In previous LAMP-based assays, single-copy genes were usually chosen as the target, such as *gyrB* (Boehme et al., 2007), *sdaA* (Joon et al., 2019), and so on. Although the relatively good sensitivity was achieved, the multi-copy elements (e.g. IS*6110*, IS*1081*) showed superior advances in the MTBC detection, and was also adopted by next generation product Xpert MTB/RIF Ultra (Charkravorty et al., 2017; Dorman et al., 2018). IS*6110* was a good molecular marker for MTBC detection because of its highly conserved and multiple copies in MTBC (Narayanan, 2004). However, some MTBC strains with low copy or missing IS*6110* have been reported (McEvoy et al., 2007). To overcome the disadvantage posed by IS*6110*-based diagnostic methods, IS*1081* was selected as a subsidiary marker in our report because it was also multiple copy elements specific in MTBC strains with stable 5 to 6 copies (van Soolingen et al., 1992). In this study, multiplex LAMP-LFB successfully detected as low as 10 fg of MTBC genomic DNA, the analytical sensitivity was higher than single-gene based LAMP assays (Kaewphinit et al., 2013). Furthermore, the high specificity was also verified as no cross-reaction with various non-MTBC strains. Therefore, combination of IS*1081* and IS*6110* could guarantee the high sensitivity and specificity among prevalent MTBC strains.

The performance of multiplex LAMP-LFB was preliminarily verified in clinical samples with comparison to Xpert MTB/RIF and culture. Multiplex LAMP-LFB (82.0%, 82/100) demonstrated higher sensitivity than both culture (47.0%, 47/100, P<0.001) and Xpert MTB/RIF (54.0%, 54/100, P<0.001) for TB diagnosis. The sensitivity of multiplex LAMP-LFB was 91.5% (43/47) in culture-positive TB cases. It is higher than that reported by a recent meta-analysis, which evaluated the accuracy of TB-LAMP on sputum samples for diagnosis of pulmonary TB and found TB-LAMP had moderate sensitivity of 77.7% and high specificity of 98.1% when using culture-based reference standard (Shete et al., 2019). Importantly, multiplex LAMP-LFB detected 28 probable TB cases, which led to the percentage increase of definite TB from 62.0% (62/100) to 90.0% (90/100). In addition, the specificity of multiplex LAMP-LFB assay reached 97.7%. Thus, the rapid and reliable confirmation of TB diagnosis will speed up the initiation of appropriated treatment.

There are several techniques that can achieve multiplex detection of IS*6110* and IS*1081* simultaneously. Xpert MTB/RIF Ultra assay integrates nest PCR to amplify IS*6110* and IS*1081*, which significantly improve the sensitivity (LOD 15.6 CFU/mL) (Charkravorty et al., 2017). But the expensive device and kit limited its use in resource limited areas. Multiple cross displacement amplification (MCDA), previously established by our group, can also perform multiplex amplification (Jiao et al., 2019). It needs 10 primers for each target, which increase the difficulty to avoid cross reactions between primers and optimize the reaction temperature for multiplex system. While LAMP approach established in this study exhibited high sensitivity and specificity in detecting MTBC and easy to perform. It is more suitable for the resource limited settings.

There are some limitations in our study. It is shown that the analytical sensitivity of multiplex LAMP-LFB mainly depends on the detection of IS*6110*. And the reference strain H37Rv used to evaluate the analytical sensitivity has 16 copies of IS*6110.* Thus, the multiplex LAMP-LFB is especially suitable for detection of MTBC strains with high copy number of IS*6110*, such as strains prevalent in China (7-18 copies) (Cai et al., 1999). But the situation might be different in other areas. It is reported that only 56% of the MTBC strains isolated in India are IS*6110*-high-copy-number strains (6-19 copies), while 20% were IS*6110*-low-copy-number strains (1-2 copies) and 11% were IS*6110*-missing strains (Chauhan et al., 2007). The LOD of multiplex LAMP-LFB in IS*6110*-low-copy-number or IS*6110*-missing strains might be higher than 10 fg. However, we did not evaluate it as none of such strains were available in our lab. Further study using large number of strains from different regions is needed to comprehensive assessment of clinical feasibility of multiplex LAMP-LFB assay.

In conclusion, we established and validated multiplex LAMP-LFB assay simultaneously targeting IS*6110* and IS*1081* for MTBC detection in pure cultures and clinical samples. Multiplex LAMP-LFB is a useful tool for diagnosis of TB, especially in probable TB cases and resource limited settings. Future studies including different kinds of samples (pleural fluid, cerebrospinal fluid, stool, etc.), target population (children, HIV-infected persons, etc.) and isolation regions (IS*6110*-low-copy-number strains or IS*6110*-missing strains prevalent areas, etc.) will be needed to further evaluate the feasibility of LAMP-LFB.

## Data Availability Statement

The original contributions presented in the study are included in the article/supplementary material. Further inquiries can be directed to the corresponding authors.

## Ethics Statement

The studies involving human participants were reviewed and approved by Beijing Chest Hospital Beijing Children’s Hospital. The patients/participants provided their written informed consent to participate in this study.

## Author Contributions

XW, WJ, and AS conceived and designed the experiments and wrote and revised the manuscript. XW, YW, and SQ performed the most experiments and analyzed the data. GW, HQ, LS, CS, and HH enrolled the subjects, collected samples, and provided reagents and materials. All authors contributed to the article and approved the submitted version.

## Funding

The study was supported by the grant from National Science and Technology Major Project of China (2018ZX10103001-003 and 2018ZX10101004-002-005), Capital’s Funds for Health Improvement and Research (2018-4-1142) and Chinese grant of NOVA Program from Beijing Municipal Science & Technology Commission (Z161100004916079).

## Conflict of Interest

The authors declare that the research was conducted in the absence of any commercial or financial relationships that could be construed as a potential conflict of interest.
